# Epigallocatechin-3-Gallate, an Active Green Tea Component to Support Anti-VEGFA Therapy in Wet Age-Related Macular Degeneration

**DOI:** 10.3390/nu15153358

**Published:** 2023-07-28

**Authors:** Janusz Blasiak, Jan Chojnacki, Joanna Szczepanska, Michal Fila, Cezary Chojnacki, Kai Kaarniranta, Elzbieta Pawlowska

**Affiliations:** 1 Department of Molecular Genetics, Faculty of Biology and Environmental Protection, University of Lodz, 90-236 Lodz, Poland; 2 Department of Clinical Nutrition and Gastroenterological Diagnostics, Medical University of Lodz, 90-647 Lodz, Poland; jan.chojnacki@umed.lodz.pl (J.C.); cezary.chojnacki@umed.lodz.pl (C.C.); 3 Department of Pediatric Dentistry, Medical University of Lodz, 92-217 Lodz, Poland; joanna.szczepanska@umed.lodz.pl (J.S.); elzbieta.pawlowska@umed.lodz.pl (E.P.); 4 Department of Developmental Neurology and Epileptology, Polish Mother’s Memorial Hospital Research Institute, 93-338 Lodz, Poland; michal.fila@iczmp.edu.pl; 5 Department of Ophthalmology, University of Eastern Finland, 70210 Kuopio, Finland; kai.kaarniranta@kuh.fi; 6 Department of Ophthalmology, Kuopio University Hospital, 70210 Kuopio, Finland

**Keywords:** age-related macular degeneration, AMD, vascular endothelial growth factor, VEGF, anti-VEGF therapy, neovascularization, green tea, epigallocatechin-3-gallate, EGCG, autophagy

## Abstract

Age-related macular degeneration (AMD) is a largely incurable disease and an emerging problem in aging societies. It occurs in two forms, dry and wet (exudative, neovascular), which may cause legal blindness and sight loss. Currently, there is not any effective treatment for dry AMD. Meanwhile, repeated intravitreal injections with antibodies effective against vascular endothelial growth factor A (VEGFA) slow down wet AMD progression but are not free from complications. (-)-Epigallocatechin-3-gallate (EGCG) is an active compound of green tea, which exerts many beneficial effects in the retinal pigment epithelium and the neural retina. It has been reported to downregulate the *VEGFA* gene by suppressing its activators. The inhibition of mitogen-activated protein kinases 1 and 3 (MAPK1 and MAPK3) may lie behind the antiangiogenic action of EGCG mediated by VEGFA. EGCG exerts protective effects against UV-induced damage to retinal cells and improves dysfunctional autophagy. EGCG may also interact with the mechanistic target rapamycin (MTOR) and unc-51-like autophagy activating kinase (ULK1) to modulate the interplay between autophagy and apoptosis. Several other studies report beneficial effects of EGCG on the retina that may be related to wet AMD. Therefore, controlled clinical trials are needed to verify whether diet supplementation with EGCG or green tea consumption may improve the results of anti-VEGFA therapy in wet AMD.

## 1. Introduction

Age-related macular degeneration (AMD) is an eye disease affecting the macula, a small oval structure in the central retina. The disease can lead to the worsening of central vision [1]. It occurs in two forms, as dry (atrophic) or wet (exudative, neovascular) AMD. Both forms may lead to blindness and sight loss, but the progression of wet AMD is much quicker than its dry counterpart if not properly treated. Several therapeutic strategies are considered to slow down wet AMD progress, but at present, the only treatment applied in clinical practice targets vascular endothelial growth factor A (VEGFA) [2]. However, anti-VEGFA therapy does not result in vision improvement and may be associated with some complications, including inflammation, hemorrhages, and retinal detachment resulting from repeated intravitreal injections [3].

Unhealthy diet is among the most consistently evidenced AMD risk factors [4]. Consequently, AMD patients are advised to consume a low-glycemic-index diet, increase their intake of dark, green, leafy vegetables, and consume fish at least twice a week, as an inverse relationship between these dietary recommendations and the risk of AMD development and progression has been reported in many studies [5,6,7,8,9]. Importantly, such dietary interventions may lower the AMD risk of carriers of genetic variants associated with AMD to the levels of non-carriers [10].

Nutrition may modulate the action of therapeutic drugs as it may change ingestion, digestion, absorption, metabolism, the functional use/activation of dependent systems, and the excretion of such drugs [11]. Therefore, pharmaceutical strategy should consider whether nutrients to be used are beneficial, detrimental, or neutral for both the disease and the therapeutic strategy. The components of green tea extract are known to exert many health-related beneficial effects in vivo and in vitro, but there is a deficit of clinical trials on the preventive potential of these agents in AMD [12]. If they are to be used to support anti-VEGFA therapy in wet AMD, they must also exert beneficial, or at least neutral, effects on the eye, especially the retina.

In this narrative/perspective review, we present some information on wet AMD and anti-VEGFA therapy. The basic aspects of VEGFA structure and regulation of its expression and activity are also presented. Finally, we present the results of studies that directly or indirectly support the use of (-)-epigallocatechin-3-gallate, a green tea component to support the anti-VEGFA therapy in wet AMD.

## 2. Wet AMD and Anti-VEGFA Therapy

Age-related macular degeneration is an eye disease affecting the macula, a small circle-like structure in the central part of the retina, which is responsible for central vision and perceiving fine details. Central vision is needed to read, watch TV, drive, and recognize faces. Advanced AMD leads to legal blindness and sight loss. This disease affects a substantial proportion of the aging population, and therefore, it is an emerging burden for societies and inflicts serious physical and mental problems on affected individuals. However, despite these serious challenges, the pathogenesis of AMD is poorly known, limiting its therapeutic options, and consequently, AMD is considered to be a largely incurable disease.

AMD may occur in two forms: dry and wet. In most AMD cases, the disease is initiated in its dry form, and in 5–25% patients, it progresses to the wet form [13]. However, the exact relationship between these two forms of AMD is not fully known, though recent studies suggest that persistent central vitreomacular adhesion (VMA) occurs in both dry and wet AMD [14]. The dry form is more common and progresses slower than its wet counterpart, but its advanced degenerative stage, manifesting as geographic atrophy (GA), characterized by the loss of retinal pigment epithelium (RPE), photoreceptors, and choriocapillaris in the macula, may lead to irreversible vision loss [15,16].

**Figure 1:**
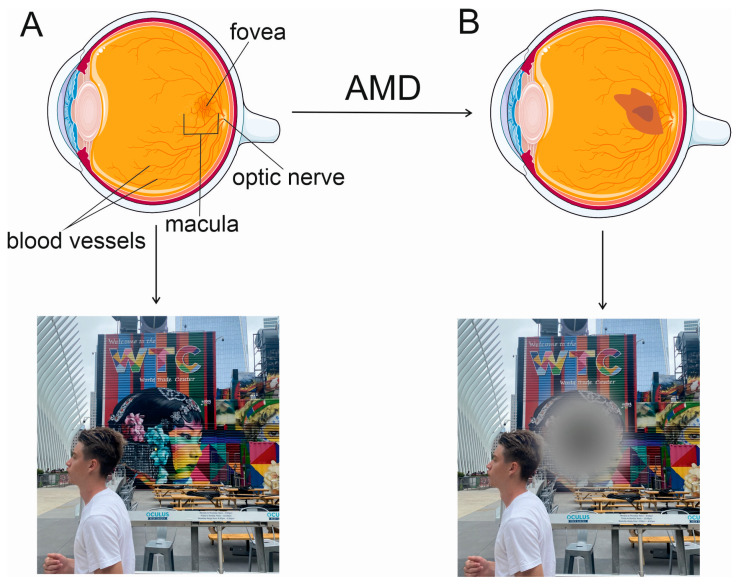
Advanced age-related macular degeneration (AMD) leads to disturbances in central vision. AMD affects the macula, a small structure in the retina, centered by the fovea (**A**). Advanced AMD occurs in two basic forms: dry (atrophic, not presented here) and wet (advanced neovascular, (**B**)). In eyes with wet AMD, the subretinal hemorrhage (dark brown) is adjacent to a choroidal neovascular membrane (light brown) and results in disturbances in or loss of central vision.

Wet AMD is characterized by choroidal neovascularization (CNV) membranes, new blood vessels that grow underneath the retina (Figure 1). The new vessels sprout from choriocapillaris (CC) through the Bruch’s membrane into the subretinal space. Wet AMD may be initiated by the loss of choroidal vasculature, underlined by a reduction in blood supply induced by the stenosis of large vessels. Such an environment acquires proinflammatory properties when AMD progresses [17]. The retinal pigment epithelium, unlike in advanced dry AMD, remains intact and produces angiogenic factors, including VEGFA, which, along with its receptors, is critical for the formation of new vessels from the CNV membranes [18]. The newly formed vessels are fragile and leak their content into the layers of the retina. This may support fibrosis, causing the development of a late-state disciform scar and irreversible vision loss if not treated.

During the process of CNV, the choroid experiences ischemia and hypoxia, inducing the expression of hypoxia inducible factor 1 subunit alpha (HIF1A), which plays an important role in the transcription of the *VEGFA* gene [19,20,21]. VEGFA signaling, critical in the formation of CNV membranes, is mediated by the VEGFA receptor 2 (VEGFR2) on the surface of the endothelial cells [22].

At present, dry AMD is incurable, but the treatment of wet AMD patients with intravitreal injections of antibodies or fusion proteins effective against VEGFA and its receptor has become the golden standard and has significantly improved the outcomes of AMD therapy [23]. Anti-VEGFA treatment may stop the worsening of visual acuity in wet AMD patients and prevent or delay vision loss. In some cases, researchers have observed not only the stabilization of visual acuity after anti-VEGFA treatment but its improvement [24,25]. Although anti-VEGFA therapy is not efficient in some patients and some individuals do not tolerate it, nowadays it is the only treatment for wet AMD that has consistently evidenced efficacy and is routinely used in a clinic setting. Therefore, efforts are being made to improve its efficiency and tolerability that depend on the susceptibility of wet AMD patients to this kind of therapy, which is determined by individual genetic constitution and environmental conditions. However, the genetic constitution is only a base for gene expression, which is largely regulated by epigenetic mechanisms, involving DNA methylation, histone modifications, and regulatory RNAs. The cellular epigenetic pattern depends on nutrition and so does the susceptibility of patients to a therapy [26]. Many studies have reported that resistance to therapy as well as its effectiveness and side effects are dependent on nutrition, and dietary recommendations are issued in the treatment of many diseases, including cancer and diseases of the immune system, nervous system, and the gastrointestinal tract, making diet an important element of personalized medicine [27,28,29,30,31].

The first anti-VEGFA drug approved by the FDA was pegaptanib, but it was replaced by other, more effective drugs [32]. Ranibizumab is a monoclonal antibody fragment effective against VEGFA, whose effectiveness and safety in wet AMD were confirmed in two large clinical trials [33,34]. Bevacizumab, a monoclonal antibody effective against VEGFA, may be a cheaper alternative for ranibizumab, but its use in the eye is not regulated by the FDA and is used off label to treat AMD, but its efficacy and safety is comparable with ranibizumab [35]. The third drug approved by FDA was aflibercept [36]. The newest anti-VEGF drugs used in clinics are brolucizumab and faricimab [13]. Regimes of injections of these anti-VEGFA compounds depend mainly on the ophthalmologist’s experience [37]. Anti-VEGFA biosimilars such as conbercept are widely used in Asia [38].

Intravitreal anti-VEGFA therapy may be associated with adverse side effects that are mainly local [39]. Endophthalmitis belongs to the most devastating ocular complications of anti-VEGFA therapy, occurring in up to 0.1% of cases [40]. Other symptoms include intraocular inflammation, an increase in intraocular pressure, rhegmatogenous retinal detachment, and ocular hemorrhage. The systemic adverse effects of anti-VEGFA therapy are very rare [41,42]. Due to repeated intravitreal injections and local complication risks, any dietary intervention to support anti-VEGFA therapy should consider the potential side effects and not to interfere with them. We could not find any evidence that green tea or its extract components might be involved in the induction or potentiation of these side effects.

Resistance to anti-VEGF therapy is a common and well-recognized problem in cancer treatment [43]. However, resistance to anti-VEGF therapy in AMD is mainly associated with recurrent exudative events occurring in wet AMD patients [44]. Pharmacodynamic tolerance to anti-VEGFA therapy in wet AMD patients may result from the elevated expression of VEGFA and its receptors, changes in signal transduction, or a shift of the stimulus for CNV growth toward other growth factors [45].

## 3. Vascular Endothelial Growth Factor A

VEGFA, a protein with vascular activity, was first extracted from a fluid released by a malignant tumor [46]. The *VEGFA* gene is a member of the *VEGF* family, consisting of gene encoding placental growth factor (PGF) and four VEGF members, VEGFA, VEGFB, VEGFC, and VEGFD (https://www.genenames.org/data/genegroup/#!/group/1267, accessed on 29 May 2023). The *VEGFA* gene codes for a heparin-binding protein, functioning in the form of disulfide-linked homodimer, stimulating the migration and proliferation of endothelial cells. The gene is crucial in both physiological and pathological angiogenesis [47]. It is upregulated in many malignant tumors and plays an important role in tumor development and metastasis [48,49].

**Figure 2:**
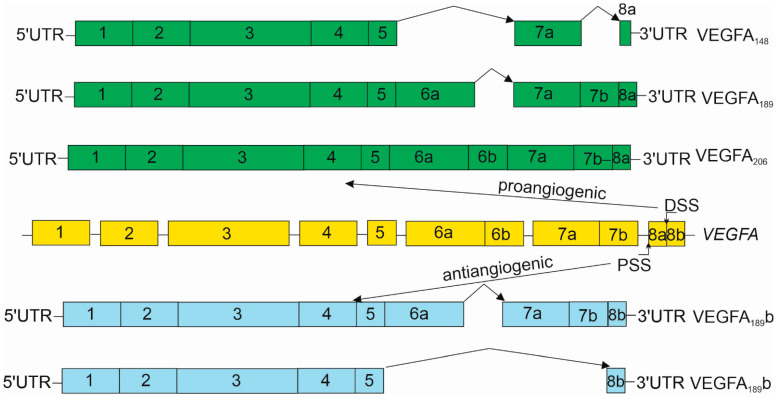
The vascular endothelial growth factor A (*VEGFA*) gene contains eight exons (yellow) separated by seven introns. Exon 8 can be divided into two parts that have alternative stop codons and alternative splicing sites—proximal (PSS) and distal (DSS). The selection of these signals results in alternative forms of the mRNAs that are translated to produce proangiogenic (green) or anti-angiogenic (blue) isoforms. The pro-angiogenic VEGFA mRNA are produced by the use of DSS, whereas their antiangiogenic counterparts use PSS. Arbitrarily selected representatives of the VEGFA isoforms are presented only. The proportions of the boxes representing exons do not necessarily correspond to the actual length of *VEGFA* exons.

The human *VEGFA* gene is located on chromosome 6 and has 16,304 base pairs (GRCh38/hg38). It has eight exons separated by seven introns (Figure 2). The first exon contains two major transcription start sites, the two translation start sites (AUG and CUG) and exon 8 has two alternative stop codons. The expression of the *VEGFA* gene produces as many as 17 isoforms due to the alternative usage of promotors, alternative splicing and alternative initiation and termination (https://www.genecards.org/cgi-bin/carddisp.pl?gene=VEGFA&keywords=vegfa#genomics-enhancers, accessed on 24 May 2023). All currently described isoforms (named according to the amino acid number of the human protein) contain exons 1–5 and two different instances of exon 8. The selection of the terminal exon splice site results in two isoform families, the proangiogenic VEGF-Axxx family and the antiangiogenic VEGF-Axxxb family. There is also evidence for alternative translation initiation from upstream non-AUG (CUG) codons resulting in additional isoforms. A C-terminally extended antiangiogenic isoform of VEGFA may be synthesized by an alternative in-frame translation termination codon and a stop codon readthrough mechanism [50]. The expression of some isoforms produced from the AUG start codon is controlled by a small upstream open reading frame located in an internal ribosome entry site [51].

The expression of the *VEGFA* gene is regulated in many ways, creating an opportunity for the production of many isoforms of the VEGFA protein that display different biological properties and result in different types of receptor binding and extra-cellular localization [52]. In addition, the stability of the VEGFA mRNA is influenced by hypoxia and growth factors via the binding of proteins at AU-rich elements in the 3′-UTR, which also contains a riboswitch that plays a role in the regulation of VEGFA mRNA translation [53,54]. The variability of the *VEGFA* gene plays a role in the functioning of its product and has been explored in studies of human diseases [55,56,57].

**Figure 3:**
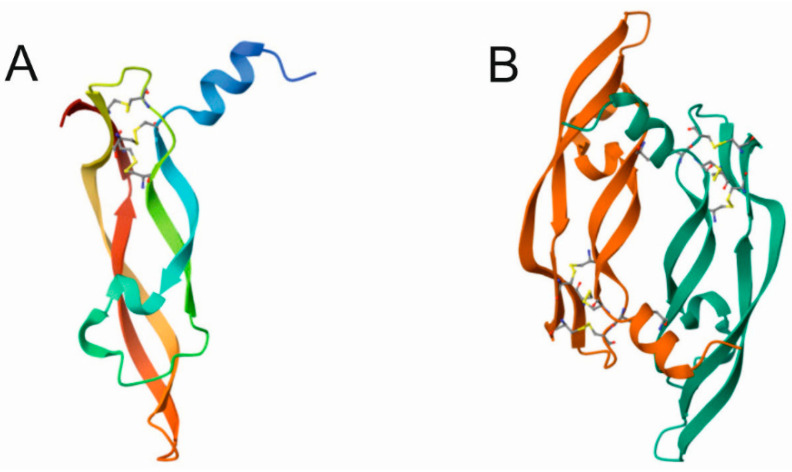
Ribbon representation of the monomer (**A**) and the dimer (**B**) of the receptor binding domain of vascular endothelial growth factor A. Disulfide bonds are represented by thin blue/yellow lines, and the two α-helices are in blue and green. The four central β-sheets form a knot motif on one end of the molecule. Taken from the RCSB Protein Data Bank: Y.A. Muller, B. Li, H.W. Christinger, J.A. Wells, B. C. Cunningham, A.M. de Vos, Vascular endothelial growth factor: crystal structure and functional mapping of the kinase domain receptor binding site (1997) https://doi.org/10.2210/pdb1VPF/pdb [59] under the CC0 1.0 Universal (CC0 1.0) Public Domain Dedication.

The overall structure of the VEGFA monomer consists of a central, antiparallel four-stranded β sheet with a cystine knot, which consists of two disulfide bonds forming a covalently linked ring structure between two adjacent β sheets [58]. VEGFA dimerizes in an antiparallel, side-by-side fashion by covalent linking two VEGFA monomers with two symmetrical disulfide bonds [59] (Figure 3).

The local concentration of VEGFA in tissues is strictly controlled in embryogenesis, resulting in its haploid insufficiency; heterozygous *VEGFA* mutants die in the early embryonal stage due to disturbances in the circulatory system [60].

**Figure 4:**
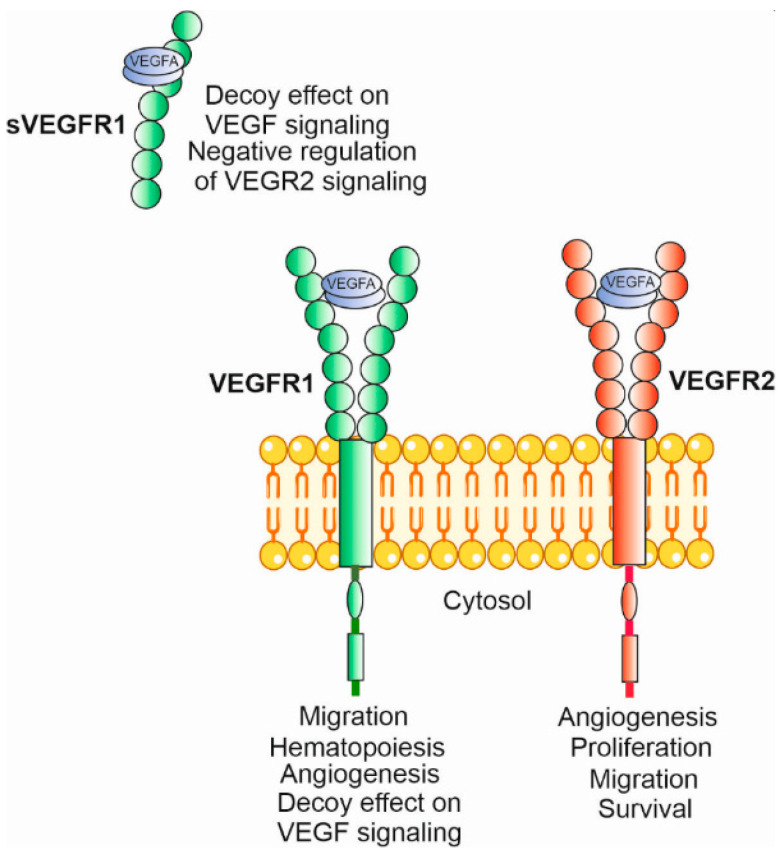
The biological activity of vascular endothelial growth factor A (VEGFA) is mediated by its receptors, VEGFR1 and VEGFR2, acting as homodimers upon the activation induced by VEGFA binding in its dimeric form. Both receptors can also act in their soluble forms and VEGFA displays an affinity to soluble VEGFR1 (sVEGFR1). Only the main biological activities of VEGFA–VEGFR complexes are indicated. VEGFR1 and VEGFR2 monomers may form a heterodimer, which also functions as a VEGFA receptor (not presented here).

The biological activity of the VEGFA polypeptide, which promotes angiogenesis, vascular permeability, cell migration, and gene expression, is mediated by VEGFA binding with its receptors, VEGFR1 and VEGFR2 (Figure 4) [18]. Therefore, these receptors can be a target of anti-VEGF therapy. Both receptors are typical tyrosine kinase receptors, with an extracellular domain for ligand binding, a transmembrane domain and a cytoplasmic domain, including a tyrosine kinase domain [61]. Although ligand binding is required for receptor dimerization and their full tyrosine kinase activity, some pre-formed VEGFR2 dimers are reported to display a certain level of such activity in vitro [62]. The affinity of VEGFA to VEGFR1 is two orders higher than to VEGFR2, but the kinase activity of the former is an order weaker than the latter [63]. VEGFR1 and VEGFR2 monomers may also form heterodimers, and VEGFR3 may also participate in the formation of heterodimers with VEGFR1 and VEGFR2 [62].

## 4. Potential of (-)-Epigallocatechin-3-Gallate to Support Anti-VEGFA Therapy in Wet AMD

Green tea is prepared from the leaves of *Camellia sinensis* and has unique nutraceutical properties [64]. It has been shown to have beneficial antioxidant, anti-inflammatory, antiaging, and antitumor effects as well as other favorable properties in different in vivo and in vitro systems [65]. Green tea extract contains catechins, polysaccharides, and flavonol, which have all been reported to have beneficial health-related features [66].

**Figure 5:**
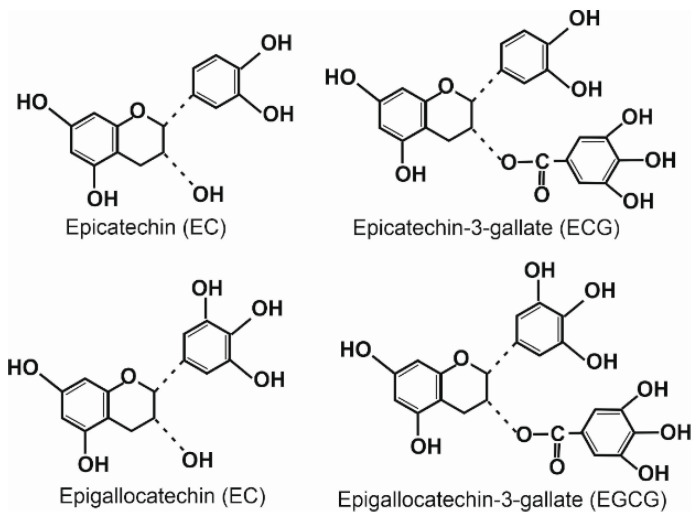
Catechins of green tea extract.

Catechins are the most intensively studied compounds of green tea extract. They include (-)-epigallocatechin-3-gallate (EGCG), (-)epigallocatechin (EGC), (-)-epicatechin-3-gallate (ECG), (-)epicatechin (EC), and (+)-catechin (Figure 5).

It is not within the remit of this paper to present all of the aspects of the beneficial action of green tea and its components in various biological systems, which can be found in many excellent recent reviews, e.g., [67,68,69,70].

The Korean Bunding AMD Cohort Study investigated the 10-year incidence of the progression of the intermediate form of AMD to its exudative counterpart in connection with some lifestyle and genetic factors [71]. Regular intake of green tea was associated with a decrease in wet AMD-related changes. However, this has been the only study to associate AMD outcome with green tea consumption. The great majority of studies are performed on cellular cultures with EGCG.

### 4.1. Angiogenesis, VEGFA and Its Receptors

Green tea extract (GTE) or its catechin, EGCG, at 40 mg/L decreased the levels of the VEGFA peptide secreted into a conditioned media of MDA-MB231 breast cancer cells and human umbilical vein endothelial cells (HUVECs) [72]. Also, the VEGFA mRNA level was inhibited on the action of GTE and EGCG in MDA-MB231 cells. This inhibition was associated with decreased activity of the *VEGFA* gene. Further analysis showed that GTE decreased the levels of the *FOS* (fos proto-oncogene, AP-1 transcription factor subunit) and *JUN* (jun proto-oncogene, AP-1 transcription factor subunit), indicating that activator-protein-AP-1-responsive regions that are in the human *VEGFA* promoter may be involved in that suppressive effects of GTE. The expression of another modulator of the *VEGFA* transcription, protein kinase C (PKC), was inhibited by GTE in MDA-MB231 cells [72].

It was shown that 30 µM EGCG inhibited the activation of mitogen-activated protein kinases 1 and 3 (MAPK1 and MAPK3) in colon cancer cell line HT29 [73]. Interestingly, the remaining catechins of green tea extract, EGC, ECG, and EC, had no effect at this concentration. MAPK1 and MAPK3 are known to induce VEGFA by serum withdrawal, and consequently, EGCG inhibited VEGFA expression in serum-deprived HT29 cells. The cells transfected with promoter–reporter constructs showed enhanced activity of the *VEGFA* promoter, secondary to serum starvation, but the treatment of the cells with EGCG inhibited the promoter activity in a dose-dependent manner. EGCG decreased both the volume and weight of a tumor after the subcutaneous implantation of HT29 cells into mice, and that decrease was as high as 58–61% at 22 days after implantation. EGCG treatment reduced the number of tumor vessels by about 1/3 as compared with controls and decreased the proliferation of tumor cells by the same amount. Finally, EGCG caused an almost twofold increase in tumor cell apoptosis and a threefold increase in endothelial cell apoptosis, as compared with those of controls. Although that work addressed the beneficial effects of EGCG in the treatment cancer, it demonstrated the important capacities of EGCG to influence angiogenesis, cell proliferation, and apoptosis, which are important in wet AMD pathogenesis. The key event observed in that work was the inhibition of MAPK1 and MAPK3 by EGCG and the resulting suppression of VEGF expression. The mechanism behind this effect requires further study, but EGCG may modulate the action of kinases required for MAPK1 and MAPK3 phosphorylation [74]. This modulation may underline the ability of EGCG to chelate metal ions, as some kinases require divalent cations for their activation [75,76].

EGCG has been shown to inhibit VEGFA-induced DNA synthesis, cell proliferation, the autophosphorylation of VEGFR1 and VEGFR2, the phosphorylation of MAPK1 and MAPK3, the mRNA expression of the early growth response 1 (EGR1), VEGF-induced intracellular signaling, and the mitogenesis of HUVECs [77]. However, another compound of green tea extract, epicatechin (EC), has not been found to induce such effects.

The cadherin–catenin adhesion system is involved in cell recognition, differentiation, and growth and the migration of the capillary endothelium [78]. In one study, it was shown that EGCG inhibited the tube formation of human microvascular endothelial cells (HMVECs) [79]. A similar effect was observed using antibodies effective against vascular endothelial (VE)-cadherin, an adhesive molecule that is important in vascular morphogenesis. The observed effects were mediated by the inhibition of VE-cadherin tyrosine phosphorylation and the suppression of AKT serine/threonine kinase 1 (AKT1) activation during tube formation induced by VEGF. Both VE-cadherin and AKT1 are downstream elements in VEGFR2 signaling.

EGCG is characterized by a relatively high instability and poor bioavailability [80]. Therefore, its synthetic derivatives need to be able to overcome these obstacles. A prodrug of EGCG obtained by its acetylation displayed an enhanced stability and bioavailability and showed antitumor activity [81]. A similar modified EGCG (pro-EGCG) was applied to study its antiangiogenic effects in laser-induced CNV in the retina of mice [82]. Increased levels of proteins of the HIF1A–VEGFA–VEGFR2 pathway in CNV regions was observed in that study, along with an increase in the M1-type macrophage–microglia markers. After laser exposure, increases in interleukin 6 (IL6) and tumor necrosis factor (TNF) were observed in the choroid-RPE complexes of the mice. Orally given pro-EGCG alleviated CNV formation in a dose-dependent manner and inhibited the production of proteins of the HIF1A–VEGFA–VEGFR2 pathway induced by CNV. The prodrug also reduced the viability, migration, proliferation, and tube formation of b-End3 cells. In conclusion, it was hypothesized that pro-EGCG might suppress HIF1A levels by inhibiting the PI3K-AKT-MTOR pathway during CNV development and in this way contribute to alleviating and reducing the CNV areas of mice by inhibiting the HIF1A–VEGFA–VEGFR2 pathway.

### 4.2. The Retinal Pigment Epithelium and the Neuroretina

It was observed that EGCG reduced the harmful consequences of ischemia/reperfusion in rats, such as a reduction in the amplitudes of a- and b-waves in the electroretinogram, a decrease in the number proteins and RNAs specific to retinal ganglion cells and photoreceptors, an increase in retinal caspases 3 and 8 mRNAs and proteins, an increase in retinal glial fibrillary acidic protein (GFAP) and its mRNA, and a decrease in optic nerve proteins associated with ganglion cell axons [83]. Also, EGCG decreased the apoptosis of retinal RGC-5 ganglion cells evoked by hydrogen peroxide. These results demonstrate that EGCG may protect retinal neurons against oxidative stress and ischemia/reperfusion.

Another study on the neural retina also showed a beneficial effect of EGCG [84]. Sodium nitroprusside (SNP), a nitric oxide donor, was used to induce oxidative stress in brain membranes, and that stress was inhibited by EGCG about 10 times more effectively than by a water-soluble analog of vitamin E (Trolox). The injection of SNP into the eyes of rats affected retinal photoreceptors, as evidenced by changes in their electroretinograms, the decrease in photoreceptor-specific markers (rhodopsin kinase), and the increase in CASP3. These effects were ameliorated by co-injection with EGCG. This study showed that not only RPE cells but also the neural retina may benefit from EGCG.

Although exposure to ultraviolet (UV) radiation may result in serious damage to all kinds of cells, the role of UV radiation in AMD pathogenesis is not completely clear [85]. The Alienor study showed that high and low exposures to ambient UV radiation might be associated with an increased risk of early AMD [86]. However, exposure to solar UV radiation induces reactive oxygen and nitrogen species (RONS) [87]. It was shown that EGCG suppressed UVA-induced ARPE-19 cell death [88]. EGCG inhibited RONS production in a dose-dependent fashion and increased the survival of the cells upon UVA exposure in a concentration-dependent manner. EGCG inhibited UVA-induced mitogen-activated protein kinases 1 and 8 (MAPK1 and MAPK8) and p38 activation. Also, EGCG inhibited the UVA-induced expression of prostaglandin-endoperoxide synthase 2 (PTGS2, COX-2). Therefore, EGCG may exert a protective effect against UVA exposure to ARPE-19 cells, contributing to a protective effect against retinal damage induced by the RONS produced by UV radiation in the eye, and the modulation of the MPK signaling pathway may lie behind this action. Similar results were obtained in a subsequent study, in which EGCG decreased UVB-radiation-induced RONS generation and apoptosis in ARPE-19 cells [70]. Although UVB does not penetrate the retina, it is used to induce AMD-resembling stress stimuli to cells and study cellular stress responses. UVB-induced apoptosis was associated with the decreased phosphorylation of MAPK8 and JUN, and this effect was partially blocked by EGCG. Therefore, ECGC may play a protective role against oxidative damage to retinal cells, and this beneficial effect is mediated by the modulation of the MAPK signaling pathway.

When the retina is in a normal state, RPE cells are quiescent, but in eyes with AMD, they can reenter the cell cycle, start proliferating and migrating, and secrete protein of the extracellular matrix [89,90]. Therefore, decreasing the ability of RPE cells to proliferate and migrate may play a role in AMD prevention and in particular, preventing the wet form of the disease [90]. It was shown that EGCG inhibited the migration of ARPE-19 cells stimulated by an isoform of platelet-derived growth factor (PDGF-BB), which plays an important role in angiogenesis by promoting the proliferation and migration of mesenchyme-derived cells [91,92]. PDGF-BB not only induced the migration of ARPE-19 cells, but also their adhesion to fibronectin. EGCG did not directly bind PDGF-BB, but it inhibited fibronectin-induced cytoskeletal reorganization. EGCG also suppressed PDGF-BB-induced PDGF-β receptors, downstream PI3K/Akt, and MAPK phosphorylation. Therefore, EGCG may inhibit RPE cell migration and adhesion to fibronectin. This is important for wet AMD pathogenesis, but may be also critical for other retinal diseases, such as proliferative vitreoretinopathy (PVR) [93,94].

Dysfunctional autophagy may contribute to AMD pathogenesis via various mechanisms (reviewed in [95]). Consequently, degradative and secretory autophagy are considered to be a therapeutic target in AMD treatment [96]. It was shown that UVB radiation induced autophagy in ARPE-19 cells, as evidenced by the expression of microtubule-associated protein 1 light chain 3 (MAP1LC3/LC3) [97]. LC3 is cleaved by autophagy-related 4 cysteine protease (ATG4) to form LC3-I, which is then conjugated with phosphatidylethanolamine to form LC3-II [98]. After incorporation into the autophagosomal membranes, LC3-II interacts with cargo receptors, which have LC3-interacting motifs (LIRs). Therefore, the amount of LC3-II may be related to the number of autophagosomes and in this way reflects autophagic flux. UVB radiation increased the LC3-II level in a dose-dependent manner, but EGCG treatment repressed UVB-mediated autophagy, as evidenced by the number of autophagosomes per cell. Further analysis showed that EGCG regulated UVB-mediated autophagy via interaction with the mechanistic target of rapamycin kinase (MTOR) signaling pathway. MTOR is the primary negative regulator of autophagy; it coordinates autophagy with cellular growth and division [99]. In one study, UVB exposure resulted in the phosphorylation of two MTOR downstream targets, suggesting the activation of the MTOR signaling pathway [100]. This was confirmed by the observation that rapamycin, an inhibitor of the MTOR pathway, abolished the formation of LC3-II mediated by UVB radiation. EGCG acted protectively against the cytotoxic effect of UVB radiation in ARPE-19 cells. EGCG treatment had no effect on the survival of cells with silenced ATG5 exposed to UVB radiation, unlike with wild-type cells, suggesting that EGCG acted on ARPE-19 cells through the modulation of autophagy.

EGCG and other catechins of green tea have been reported to modulate autophagy in many other cellular systems. Autophagy may help cells under endoplasmic reticulum (ER) stress to survive via its interplay with apoptosis and MTOR- protein kinase AMP-activated catalytic subunit alpha 1 (PRKAA1/AMPK) [101]. It was shown that EGCG promoted the autophagy-dependent survival of HEK293T cells in ER stress [102]. The observed effect was dependent on MTOR and unc-51-like autophagy activating kinase (ULK1). Studies on the beneficial effects of EGCG and the other active components of green tea mediated by autophagy mainly focus on cancer, but also address neural cells and other systems related to vision [103,104].

Retinal degeneration is also a feature of diabetic neuropathy (DR), which is characterized by high glucose (HG) concentration in the retina [105]. It was shown that EGCG stimulated autophagy by mediating the formation of autophagosomes and increasing lysosomal acidification and autophagic flux in Müller retinal cells, but HG concentration was associated with an inhibition of autophagy, underlined by the accumulation of sequestosome 1 (p62/SQSTM1) cargo, the downregulation of beclin 1 (BECN1) and an increase in apoptosis [104]. However, HG treatment of Müller cells with EGCG resulted in autophagy activation, protection from apoptosis, and increased proliferation. In a DR experimental model, EGCG reduced the retinal damage induced by HG. DR and AMD share only some elements of pathogenesis, such as retinal degeneration, but the effects observed in DR induced by HG concentration may correspond to those observed in AMD and may be associated with oxidative stress and RONS overproduction [106]. RONS may induce mitochondrial damage in retinal cells, resulting in degeneration and apoptosis, important in the AMD phenotype [107].

Retinis pigmentosa (RP) is a group of inherited retinal diseases that, similarly to AMD, lead to photoreceptor loss, but, in unlike AMD, their progression is associated with loss of peripheral rather than central vision [108]. It was shown that a rat model of RP treated intraperitoneally with EGCG exhibited better visual and retinal electrical functions, with more contrast sensitivity and higher b-wave values than controls [109]. Also, EGCG decreased lipid peroxidation and increased total antioxidant capacity and catalase (CAT) and SOD1 activities.

Apart from catechins, green tea extract also contains polysaccharides that have attracted less attention than catechins, despite their apparent beneficial health-related effects and preventive/therapeutic applications [110,111]. In one study, it was shown that a green tea polysaccharide increased cell viability, decreased apoptosis, and inhibited the production of RONS and MDA in ARPE-19 cells challenged with hydrogen peroxide [112]. In addition, the polysaccharide restored the activity of SOD1, CAT, glutathione peroxidase 1 (GPX1), and glutathione (GSH) in these cells. The anti-apoptotic action of the polysaccharide was underlined by the upregulation of BCL2-associated X, apoptosis regulator (BAX), and cleaved caspase-3 (CASP3) and a decrease in the BCL2 apoptosis regulator (BCL2) protein in ARPE-19 cells treated with H_2_O_2_. Therefore, not only catechins but also other active components of green tea may be useful in supporting retina-targeting therapies.

Although EGCG displays beneficial effects in many cellular systems, high levels of this catechin can also induce oxidative stress [113]. This was shown in a study in which rats were fed a green tea extract and catechin mixture containing different concentrations of EGCG. In this study, the pharmacokinetics, diurnal variation of oxidative status, and changes in transcription factors in ocular tissues of rats were investigated. At high EGCG concentrations, green tea extract induced an increase in oxidative stress in the plasma, aqueous humor, vitreous humor, cornea, and retina, but it decreased the stress in the lens and choroid-sclera of the animals. At low EGCG concentrations, the catechin mixture lowered the amount of 8-isoprostane in the retina and lens. The treatment induced SOD1 and GPX3 expression but suppressed CAT in the retina. Therefore, an optimal and safe concentration of EGCG should be determined when used as part of a therapeutic strategy.

## 5. Conclusions and Perspectives

Unlike its dry counterpart, wet AMD may be treated with anti-VEGF monoclonal antibodies to slow down its progression and delay possible vision loss. Wet AMD may result in serious consequences not only for the eye and vision but also for the general state of the organism [114]. We and others have showed that wet AMD patients display a greater mortality than controls, and their survival has been inversely associated with the number of anti-VEGFA injections they undergo per year [115]. Therefore, it is justified to search for modalities supporting anti-VEGFA therapy in wet AMD.

Although anti-VEGF therapy is the only commonly accepted wet AMD treatment, it does not lead to a significant improvement in visual acuity. Therefore, studies on the increase in anti-VEGFA therapy in wet AMD are justified.

In this work, we tried to show that EGCG, an active green tea component, may display several beneficial effects in various biological systems that can be related to AMD. These include vision improvements and protective action against detrimental cellular effects that may accompany AMD (Table 1). Therefore, EGCG may have the potential to exert its beneficial effects in AMD patients undergoing anti-VEGFA therapy. This is especially important as EGCG has been reported to inhibit VEGF-related angiogenesis and release of VEGFA.

**Figure 6:**
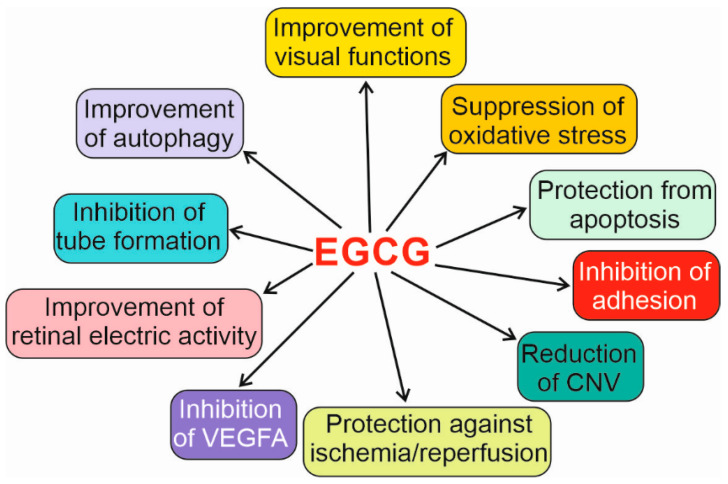
Biological effects of (-)-epigallocatechin-3-gallate (EGCG) that may improve vision and protect against the detrimental action caused by various stresses, including oxidative stress. These effects may support the therapy in fighting against vascular endothelial growth factor A (VEGFA) and its receptors, the only approved treatment of wet (exudative) age-related macular degeneration (AMD). These effects have been observed in laboratory animals and/or cell cultures. CNV—choroidal neovascularization.

Many mechanisms may lie behind the profitable effects of EGCG on the retina and therefore can be considered to support anti-VEGFA therapy (Figure 6). Mitogen protein-activated kinases 1, 5 and 8 seem to be especially important, as they may underline EGCG effects associated with angiogenesis of crucial significance in wet AMD pathogenesis and therapy.

Except for when given in extremely high concentrations, EGCG is not reported to cause any detrimental effects. We tried to show that EGCG might exert beneficial effects on retina affected by wet AMD during anti-VEGFA therapy. We do not claim that EGCG or the other components of green tea extract may be essential for such therapy but can speculate that they may increase its efficacy and/or diminish unwanted side effects. However, this is only a speculation/perspective that might be verified by perspective controlled clinical trials.

## Figures and Tables

**Table 1:** Biological effects of (-)-epigallocatechin-3-gallate (EGCG) that can be linked to age-related macular degeneration (AMD) in various biological systems.

System	Effect	Reference
MDA-MB231 breast cancer cells and HUVECs ^1^	Decreased VEGFA secreted into conditioned media	[72]
Colon cancer cell line HT29	Inhibited activation of mitogen-activated protein kinases 1 and 3 (MAPK1 and 3), increased apoptosis, and decreased angiogenesis and proliferation	[73]
HUVECs	Inhibition of VEGFA-induced DNA synthesis, cell proliferation, the autophosphorylation of VEGFR1 and VEGFR2, the phosphorylation of MAPK1 and MAPK3, the mRNA expression of EGR1, VEGFA-induced intracellular signaling, and mitogenesis	[77]
Human microvascular endothelial cells	Inhibited tube formation	[79]
Mice with laser-induced CNV, mouse b-End3 cells	Inhibited tube formation, reduced CNV area	[82]
Rats, retinal RGC-5 ganglion cells	Protection against oxidative stress and ischemia/reperfusion	[83]
Rats	Protection of the neural retina against oxidative stress	[84]
Human retinal pigment epithelium ARPE-19 cells	Suppression of UVA-induced cell death, inhibition of MAPK1, MAPK8, and p38	[88]
	Inhibition of migration and adhesion to fibronectin	[91,92]
	Repression of UVB-mediated autophagy underlined by the interaction with MTOR	[97]
Derivative of human embryonic kidney HEK293T cells	Promotion of autophagy-dependent survival in ER stress, dependent on MTOR and ULK1	[102]
Müller retinal cells	Autophagy activation and protection from apoptosis induced by high glucose concentration mediated by changes in SQSTM1 and BECN1	[104]
Rat model of RP	Improvement in visual functions, retinal electric activity, and antioxidant defense	[109]
Rats	Increased oxidative stress at high EGCG concentrations	[113]

^1^ Abbreviations: BECN1, beclin 1; CNV, choroidal neovascularization; ER, endoplasmic reticulum; EGR1, early growth response 1; HUVEC, human umbilical vein endothelial cells; MAPK, mitogen-activated protein kinase; MTOR, mechanistic target of rapamycin; RP, retinis pigmentosa; SQSTM1, sequestosome 1; ULK1, unc-51-like autophagy activating kinase; VEGFA, vascular endothelial growth factor A; VEGFR, VEGFA receptor.

## Data Availability

Not applicable.
